# Successful removal of a central venous catheter misplaced in the right subclavian artery using an intravascular stent: a case report

**DOI:** 10.1186/s40981-021-00418-y

**Published:** 2021-02-07

**Authors:** Haruka Yoshida, Shinichiro Ikemoto, Yasuyuki Tokinaga, Kanako Ejiri, Tomoyuki Kawamata

**Affiliations:** Department of Anesthesiology, Wakayama Medical University School of Medicine, 811-1 Kimiidera, Wakayama City, 641-0012 Japan

**Keywords:** Central venous catheter, Intravascular stent, Subclavian artery, Removal

## Abstract

**Background:**

Cannulation of a central venous catheter is sometimes associated with serious complications. When arterial cannulation occurs, attention must be given to removal of a catheter.

**Case presentation:**

A 62-year-old man was planned for emergency thoracic endovascular aortic repair. After the induction of anesthesia, a central venous catheter was unintentionally inserted into the right subclavian artery. We planned to remove the catheter. Since we considered that surgical repair would be highly invasive for the patient, we decided to remove it using a percutaneous intravascular stent. A stent was inserted through the right axillary artery. The stent was expanded immediately after the catheter was removed. Post-procedural angiography revealed no leakage from the catheter insertion site and no occlusion of the right subclavian and vertebral arteries. There were no obvious hematoma or thrombotic complications.

**Conclusions:**

A catheter that has been misplaced into the right subclavian artery was safely removed using an intravascular stent.

## Background

The widespread use of ultrasound has made cannulation of a central venous catheter safer [1, 2]. However, cannulation of a central venous catheter is sometimes associated with serious complications including inadvertent arterial puncture or arterial cannulation. When arterial cannulation occurs, attention must be given to removal of the catheter.

We recently encountered a case in which a central venous catheter was unintentionally inserted into the right subclavian artery. The catheter was removed by using an intravascular stent without any complications.

## Case presentation

A 62-year-old man (body weight, 80 kg; height, 185 cm) with traumatic descending aorta dissection was transferred to our hospital. He was in a state of shock (blood pressure, 75/61 mmHg; heart rate, 142 beats/min) and emergency thoracic endovascular aortic repair was planned. After entering the operating room, arterial cannulation was performed in the radial artery. Anesthesia was induced with intravenous fentanyl, propofol, and rocuronium, and his trachea was intubated. Anesthesia was maintained with air, oxygen, desflurane, and remifentanil. After the induction of anesthesia, he remained in a state of shock with systolic blood pressure of 60–70 mmHg, and common carotid artery palpation was weak. Using short-axis ultrasound imaging, a 12-gage central venous catheter (CV RegaForce EX®, Terumo, Tokyo) was inserted in the right internal jugular vein by the Seldinger technique. After catheter placement, the pressure waveform from the central venous catheter showed the arterial blood pressure waveform, indicating arterial cannulation. Since administration of heparin was planned during surgery, we decided not to remove the catheter that had been inserted into the artery. The endovascular surgery was completed in 1 h and 59 min. A postoperative chest radiograph showed the catheter in his right chest. He was transferred to the intensive care unit (ICU) without extubation.

After entering the ICU, the shock state persisted despite continuous administration of noradrenaline and dopamine. Contrast-enhanced CT showed intra-abdominal bleeding from liver injury and intestinal necrosis. It also showed that the tip of the catheter was located in the right brachiocephalic artery. A laboratory examination revealed coagulopathy: low fibrinogen level (70.0 mg/dL), prolonged PT-international normalized ratio (INR) (2.60), and prolonged aPTT (≥ 200.0 s). The catheter was therefore not removed. Two hours after entering the ICU, hepatic artery embolization, small intestine resection, and right hemicolectomy were performed.

**Fig. 1:**
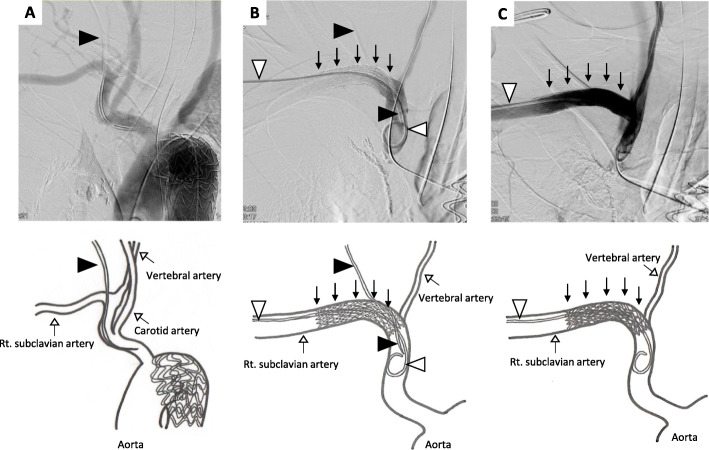
Angiography. **a** The catheter was inserted into the artery near the bifurcation of the right vertebral artery and subclavian artery. **b** An intravascular stent and a guidewire, which was inserted into the right subclavian artery. **c** An expanded stent and right vertebral artery flow. A black arrow head indicates a central venous catheter migrated into the right subclavian artery. A white arrow head indicates a guidewire for an intravascular stent. Black arrows indicate an intravascular stent. White arrows indicate corresponding arteries

On the 6th day after entering the ICU, he remained to be intubated and received noradrenaline and dopamine to maintain his blood pressure. His blood coagulability was improved: fibrinogen level, 415.0 mg/dL; PT-INR, 1.22; and aPTT, 31.2 s. We therefore planned to remove the catheter. We considered that surgical repair would be highly invasive for the patient and decided to place a percutaneous intravascular stent after removal of the catheter. Angiography showed that the catheter was inserted into the artery near the bifurcation of the right vertebral artery and subclavian artery (Fig. 1a). A stent (GORE® VIABAHN® Stent graft, Japan Gore, Tokyo) was percutaneously inserted through the right axillary artery. The position of stent deployment was adjusted so that the stent covered the catheter insertion site and did not occlude the opening of the right vertebral artery (Fig. 1b). After the catheter was removed, the stent was immediately expanded. Post-procedural angiography revealed that there was no leak from the catheter insertion site and no occlusion of the right subclavian artery and right vertebral artery (Fig. 1c). There were no obvious hematoma or thrombotic complications.

Although the catheter was successfully removed, the patient’s general condition gradually deteriorated. Thirteen days after entering the ICU, he died of multiple organ failure.

## Discussion

We encountered a case in which a central venous catheter misplaced into the right subclavian artery was safely removed using an intravascular stent. Similar cases are found in academic journals in the fields of vascular surgery and radiology [3–5], but we could not find a similar case in anesthesia-related academic journals. Therefore, we reported our case to inform anesthesiologists about the usefulness of an intravascular stent for removal of a catheter that has been misplaced into an artery.

Possible strategies for removal of a central venous catheter that has been misplaced into an artery include (1) simple removal of the catheter and application of pressure, (2) surgical repair, and (3) endovascular treatment. According to a practical guide for a central venous catheter by the Japanese Society of Anesthesiologists (http://anesth.or.jp/files/pdf/JSA_CV_practical_guide_2017.pdf), a catheter that has been misplaced into an artery can be removed if the catheter has a small diameter (2.3 mm or less) and also if compression hemostasis is possible. On the other hand, if a catheter is thicker than 7 Fr (2.3 mm or more in diameter) or if it is placed in an artery that cannot be compressed, it is recommended to consult a vascular surgeon. Removal of a catheter that has been misplaced into an artery may cause serious complications such as bleeding, hemothorax, cerebral infarction, pseudoaneurysm, and arteriovenous fistula (http://anesth.or.jp/files/pdf/JSA_CV_practical_guide_2017.pdf). Since we inserted a catheter with a diameter larger than 7 Fr into the subclavian artery, where astriction is difficult, we did not choose to simply remove the catheter and apply pressure. In addition, since our patient’s general condition had deteriorated, direct surgical repair was considered to be highly invasive for him. Therefore, we chose endovascular treatment with an intravascular stent.

A similar case in which a central venous catheter misplaced into the subclavian artery was safely removed using an intravascular stent was reported [6]. It has been reported that endovascular treatment with an intravascular stent for management of subclavian arterial injuries has a lower complication rate and a higher survival rate than those when direct surgical repair is used [7]. Endovascular treatment may also be effective for cases in which surgical repair is difficult due to anatomical reasons. Therefore, removal of a catheter using an intravascular stent is considered to be one of the treatment options, especially in cases in which astriction is difficult.

There are some precautions when using an intravascular stent: an appropriately sized stent should be chosen to prevent extravasation, and the anatomical relationship between the catheter puncture site and the surrounding major vessels should be assessed to prevent occlusion of arterial branches. It should also be noted that antithrombotic therapy is required after stent placement. In addition, since the long-term patency rate of stents is unknown, further study on the long-term efficacy will be necessary.

## Conclusion

We encountered a case in which a central venous catheter misplaced into the right subclavian artery was safely removed using an intravascular stent.

## Data Availability

Not applicable

## References

[1] Peris A, Zagli G, Bonizzoli M, Cianchi G, Ciapetti M, Spina R, Anichini V, Lapi F, Batacchi S (2010). Implantation of 3951 long-termcentralvenous catheters: performances, risk analysis, and patient comfort after ultrasound-guidance introduction. Anesth Analg.

[2] Rupp SM, Apfelbaum JL, Blitt C, Caplan RA, Connis RT, Domino KB, Fleisher LA, Grant S, Mark JB, Morray JP, Nickinovich DG, Tung A, American Society of Anesthesiologists Task Force on Central Venous Access (2012). A report by the American Society of Anesthesiologists Task Force on Central Venous Access. Practice Guidelines for Central Venous Access. Anesthesiology.

[3] Pikwer A, Acosta S, Kolbel T, Malina M, Sonesson B, Akeson J (2009). Management of inadvertent arterial catheterization associated with central venous access procedures. Eur J Endovasc Surg.

[4] Nicholson T, Ettles D, Robinson G (2004). Managing inadvertent arterial catherization during central venous access procedures. Cardiovasc Intervent Radiol.

[5] Jahromi BS, Tummala RP, Levy EI (2009). Inadvertent subclavian artery catheter placement complicated by stroke: endovascular management and review. Catheter Cardiovasc Interv.

[6] Abi-Jaoudeh N, Turba UC, Arslan B, Hagspiel KD, Angle JF, Schenk WG, Matsumoto AH (2009). Management of subclavian arterial injuries following inadvertent arterial puncture during central venous catheter placement. J Vasc Interv Radiol.

[7] Branco BC, Boutrous ML, DuBose JJ, Leake SS, Charlton-Ouw K, Rhee P, Mills JL, Azizzadeh A (2016). Outcome comparison between open and endovascular management of axillosubclavian arterial injuries. J Vas Surg.

